# Using Community Informants to Estimate Maternal Mortality in a Rural District in Pakistan: A Feasibility Study

**DOI:** 10.1155/2015/267923

**Published:** 2015-02-09

**Authors:** Ali Mohammad Mir, Mohammad Saleem Shaikh, Siti Nurul Qomariyah, Gul Rashida, Mumraiz Khan, Irfan Masood

**Affiliations:** ^1^Population Council, Islamabad 44000, Pakistan; ^2^Center for Family Welfare, Faculty of Public Health, University of Indonesia, Jakarta 16424, Indonesia

## Abstract

*Background.* We aimed to assess the feasibility of using community-based informants' networks to identify maternal deaths that were followed up through verbal autopsies (MADE-IN MADE-FOR technique) to estimate maternal mortality in a rural district in Pakistan.* Methods*. We used 4 community networks to identify deaths in women of reproductive age in the past 2 years in Chakwal district, Pakistan. The deaths recorded by the informants were followed up through verbal autopsies.* Results*. In total 1,143 Lady Health Workers (government employees who provide primary health care), 1577 religious leaders, 20 female lady councilors (elected representatives), and 130 nikah registrars (persons who register marriages) identified 2001 deaths in women of reproductive age. 1424 deaths were followed up with verbal autopsies conducted with the relatives of the deceased. 169 pregnancy-related deaths were identified from all reported deaths. Through the capture-recapture technique probability of capturing pregnancy-related deaths by LHWs was 0.73 and for religious leaders 0.49. Maternal mortality in Chakwal district was estimated at 309 per 100,000 live births.* Conclusion*. It is feasible and economical to use community informants to identify recent deaths in women of reproductive age and, if followed up through verbal autopsies, obviate the need for conducting large scale surveys.

## 1. Background

Pakistan is one of six countries that account for more than 50% of the world's maternal deaths [1]. According to Population Council estimates, each year, nearly 8.6 million women become pregnant in Pakistan. Of these, 15%, that is, 1.2 million women are likely to face obstetric complications. Each year, there are nearly 14,000 pregnancy-related deaths (PRDs). According to the Pakistan Demographic and Health Survey (2006-07) Pakistan's MMR is 276 per 100,000 live births [2].

Improving maternal health is one of the health-related Millennium Development Goals (MDGs) and targets a three-quarter reduction in the maternal mortality ratio (MMR) between 1990 and 2015 [3]. Pakistan has been identified among the 75 priority countries by the “Countdown to 2015 Initiative” where an accelerated action on maternal, newborn, and child health is required.

The Government of Pakistan has, in recent years, initiated a number of major projects to improve maternal health outcomes in the country. In 2005, it launched a comprehensive maternal, newborn, and child health (MNCH) program that aimed at improving access to Skilled Birth Attendance by employing a new cadre of Community Midwives (CMWs)—rural women who belong to the same community as their clients.

While maternal mortality has fallen from 533 per 100,000 live births (NIPS) in 1990-91 to 276 in PDHS 2006-07, Pakistan is unlikely to achieve its MDG target of reducing maternal mortality to 140 per 100,000 live births [4]. To assess how far its efforts have been successful in terms of improving access to community-based and facility-level interventions, it is important that recent estimates of maternal mortality are made available to policymakers and program managers.

Although the MMR is accepted as an important development indicator at the international and national levels, the range of simple, reliable, and feasible methods for measuring maternal mortality is still limited, especially in developing countries. Maternal mortality is difficult to measure for a number of reasons. First, maternal death is a rare event and difficult to capture; large samples are needed for it to be reliable. For the same reason, it is also expensive to gauge. Second, at present there is no standard method that can be universally applied for measuring mortality. According to the World Health Organization (WHO), identifying maternal deaths requires three elements: identifying (i) all deaths of women of reproductive age (WRA), (ii) their pregnancy-related status, and (iii) the cause of death. Without complete vital registration systems and certification of the cause of death, all three components are difficult to measure accurately [5, 6].

A vital registration system can most accurately estimate maternal mortality if the system includes questions on pregnancy-related status and cause of death. However, even where coverage is complete and all deaths medically certified, maternal deaths are frequently missed or misclassified in the absence of active case-finding [7].

Few developing countries have a vital registration system that ensures sufficient coverage and quality to enable it to serve as the basis for assessing levels and trends in cause-specific mortality, including maternal mortality [8]. Instead most developing countries rely on household surveys that require large samples to produce reliable estimates with reasonable confidence intervals [6].

In Pakistan, maternal mortality estimates have largely been derived from household surveys. The Pakistan Demographic and Health Survey (2006-07) [2] was the only nationally representative survey that provided the MMR estimate of 276 per 100,000 live births.

Keeping in mind the wide diversity among the four provinces of Pakistan regularly updated maternal mortality estimates, especially at the provincial and district levels, are now required to plan new initiatives, to monitor and evaluate existing MNCH programs, and to introduce greater accountability. This information can also help in advocacy efforts to increase awareness about maternal health issues among the public and increase the focus of policymakers on this neglected area and maintaining pressure towards achieving the MDGs and the new goals beyond 2015.

Most recently, new techniques using village informants to provide information on health-related events have been introduced in many countries, including Cambodia, Indonesia, and India [9–11]. A study carried out in India shows that through community enquiries, maternal deaths were identified by multiple informants and investigated by a doctor leading to an estimation of maternal mortality at 230 per 100,000 live births. The study showed that retrospective inquiry from multiple sources in the community is a rapid, simple, and low cost method to estimate the level and pattern of maternal mortality in countries where registration of vital events is grossly incomplete and investigation of causes is unsatisfactory [12]. A similar method was employed in a research study using key community informants to estimate maternal mortality in Kabul, Afghanistan; Mae La camp for Karen refugees, Thai-Burma border; Chiradzulu District, Malawi; and Lugufu and Mtabila refugee camps, Tanzania. It was estimated that the informant method required an average of 29% less time inputs and 33% less financial inputs in all four study sites when compared with retrospective surveys within a 6-month recall period [13]. A study piloted in Tigray, Ethiopia, used an identical approach where Traditional Birth Attendants (TBAs), community-based health workers, and midlevel providers (including nurses and nurses-midwives) were trained to obtain timely data on maternal mortality and distribution by cause of death [14] In Tanzania, a district level surveillance system was successfully implemented using a network of community leaders complemented by verbal autopsy questionnaires assessed by a panel of physicians [15].

Another community informant based approach entitled “Maternal Death from Informants/Maternal Deaths Follow-On Review (MADE-IN/MADE-FOR)” has been developed by the Initiative for Maternal Mortality Programme Assessment (Immpact project) at the University of Aberdeen, UK. It enables the measurement of maternal mortality down to the community level, together with an analysis of the causes of maternal deaths. It is also less costly than household surveys, especially in lower-fertility, lower-mortality contexts. The approach goes beyond simply counting deaths—it also develops an understanding of why they happened and how they could have been averted.

The MADE-IN/MADE-FOR technique has, so far, been successfully applied by Immpact in two districts of Indonesia [9]. Unlike some alternative methods, such as the sisterhood method, MADE-IN/MADE-FOR allows recording of all maternal deaths in a defined area, enabling more precise estimates of maternal mortality in relatively small populations [16]. It also raises community awareness of maternal health issues and acts as an advocacy tool. Its limitations include the possible underreporting of sensitive deaths, such as those related to abortion, and overlooking of early-pregnancy deaths. The approach, however, relies on the availability of existing networks within a community. Community-based approaches to identify maternal deaths have also been applied previously.

In this paper, we discuss the feasibility of applying this technique in one district in Pakistan in terms of identifying networks and their ability to identify deaths in women of reproductive age, their potential to ascertain the pregnancy-related status of the deaths, and the potential to scale up the approach.

## 2. Methods

### 2.1. Study Design and Participants

The study was conducted in Chakwal district—a predominantly rural district with only 14% urban population (total population 1.4 million). Skilled Birth Attendance (SBA) coverage in the district is 75% [17]. Administratively it is divided into 4 tehsils (subdistricts), namely, Choa Saidan Shah, Talagang, Kalar Kahar, and Chakwal. It has 3 secondary tertiary care health facilities and 74 primary health care facilities (the district has 68 union councils—the smallest administrative unit of government). The study was conducted in all 68 union councils. The total duration of the study was from November 2013 to March 2014.

### 2.2. Ethical Approval

Ethical approval was obtained from the National Bioethics Committee of Pakistan.

### 2.3. Calculating Live Births

We used existing data sources such as age specific fertility rates and calculated the number of live births.

### 2.4. Phases of the Study

#### 2.4.1. Identifying Networks

Working closely with key stakeholders including government policy makers, researchers, and program managers, we identified, from a range of possible informant networks, four networks who could provide reliable information. These included the Lady Health Workers (LHWs) network that comprises village based primary healthcare providers. Each LHW serves a population of 1000. She has to register and regularly visit 200 homes in her catchment area. The LHWs network is available throughout the country; however, it covers only 60% of the population. Religious leaders (Village Imaams) are universally available and well reputed members of the community. They lead funeral prayers and they are knowledgeable about deaths in communities. Elected councilors and nikah registrars who record marriages have access to information based on their standing in their respective communities and the nature of the work that entails frequent interaction with community members.

#### 2.4.2. Assembling the Networks (MADE-IN)

The secretary of the union council (a local government official) invited the informants to preparatory meetings that were held at the office of the union council and health facilities. Separate meetings were held for each network in two steps, that is, preparatory meetings and listing meetings. During the preparatory meetings, informants were briefed on the purpose of the activity. Keeping in view ethical considerations, the risks and benefits of voluntary participation in this activity were stressed. A detailed briefing on the maternal health situation in Pakistan, the Islamic injunctions on saving innocent lives, and the special status accorded to mothers in a Muslim society were stressed. Informed consent for participation in the study was obtained from all informants. This helped in convincing the informants to participate in the study on a voluntary basis. Orientation was also provided on filling in the WRA death listing form. Informants were required to collect information related to all deaths in women within the age group 12–50 years who had died in the preceding two years, that is, January 2012 to December 2013. They were asked to use the forms to document the following information: whether the women had died during pregnancy, delivery, or within six weeks postpartum and abortion; the date and place of death; age, name of husband, and residential address.

There were no refusals for attending the listing meetings by the LHWs, nikah registrars, and lady councilors. Only 3 percent of the religious refused to participate.

#### 2.4.3. Follow-Up of Deaths (MADE-FOR)

All reported deaths were then followed up through home visits, where detailed information was obtained on each death from the next of kin, using the revised WHO VA questionnaire with additional questions on the socioeconomic characteristics of the family, health-seeking behaviour, and quality of care offered at the health facility. The VAs were conducted with the deceased's next of kin. For the verbal autopsies the refusal rate was 1.2 percent.

Subsequently, the VA forms were analyzed to determine the cause of deaths using a computerized algorithm (InterVA-M software) [18] that is compatible with the WHO questionnaire. Together, these two steps—MADE-IN and MADE-FOR—provided village-level estimates of the number of WRA deaths and in particular the number of PRDs and maternal deaths in a defined period. A PRD is defined as “the death of a woman while pregnant or within 42 days of termination of pregnancy, irrespective of the cause of death” [18]. WHO defines maternal death as “the death of a woman while pregnant or within 42 days of termination of pregnancy, irrespective of the duration and site of pregnancy, from any cause related to or aggravated by the pregnancy or its management but not from accidental or incidental causes.” The definition implies the inclusion of maternal deaths from either direct or indirect obstetric causes of death [19].

#### 2.4.4. Estimating the Probability of Capturing Deaths by the Informant Networks

In tehsils where both LHWs and religious leaders' networks were used, we were able to identify whether the LHWs or religious leaders or both had identified deaths. This enabled us to apply the capture-recapture technique to estimate both the total number of PRDs in these tehsils and the coverage of each network, that is, the proportion of the total deaths identified by each network. Capture-recapture analysis is used in epidemiology to assess the completeness of reporting by analyzing the overlap among different lists of events under investigation [20, 21].

Collectively, the four networks identified 2,001 WRA deaths during the period 2012-13. The next step was to conduct VAs with the deceased women's relatives, in most cases, their siblings, in-laws, parents, other relatives, or spouse.

## 3. Results

Figure 2 shows the distribution of respondents with whom VAs were conducted. Only 1,808 respondents, that is, relatives of the deceased, could be contacted for a VA; after autopsy, we found that, in 167 cases, the deaths had not taken place in the last two years. In 97 cases, the address was found to be a duplicate while 23 respondents could not be located as the address provided was incomplete. In 278 cases, the reported deaths were not within the specified age band of 12–50 years and hence were excluded. 12 respondents refused to participate. In total, 1424 women deaths met the criteria for inclusion in the study.

### 3.1. Probability of Each Informant Network Capturing Deaths

In the union councils of Chakwal where both the LHWs and religious leaders' networks were used to capture eligible PRDs (i.e., within the set period and location), 72 eligible PRDs were identified by both networks. Of these, 62 cases were identified by the LHWs network, 38 by the religious leaders' network, and 28 by both networks. A simple formula was used to estimate the total number of cases: *T* = *N*1 × *N*2/*M*, where *N* is the number of cases captured by LHWs or religious leaders and *M* is the number of cases captured by both networks.

We calculated the estimated number of total PRD cases occurring in Chakwal tehsil by applying the capture-recapture technique which gave us a figure of 84. Please see Figure 3.

In Talagang, where again both networks were used, a total of 45 cases were recorded. Of these, 37 were recorded by LHWs, 26 by religious leaders, and 18 by both. We estimated the probability of an eligible PRD being identified by applying the capture-recapture formula in Talagang to be 53.

The probability of an eligible PRD being identified by the LHWs network in Chakwal was 0.73 (obtained by dividing the number of deaths identified by a network by the adjusted number of deaths obtained from the capture-recapture formula as seen above); in Talagang it was 0.45. The probability of religious leaders capturing deaths in Chakwal was 0.45 while in Talagang it was 0.49.

To estimate the number of total deaths in the tehsils of Kallar Kahar and Choa Saidan Shah, where only single network was used, we adjusted the number on the basis of the probability of LHWs reporting deaths in Chakwal, which is 73%.

The four networks captured in total 169 PRDs. By applying the capture-recapture technique, the adjusted number of deaths that were captured was estimated to be 186. Therefore, the probability of reporting deaths through this study is estimated to be (169/186 × 100) = 91% (95% CI 85%–95%).

Based on the pilot dataset, once all identified WRA deaths had been verified and the VAs performed, we found that the village informants had incorrectly classified 3 out of 169 eligible PRDs as not being eligible. On the other hand, 18 out of the 169 deaths were identified as eligible PRDs while they were actually false matches.

Using VAs to confirm the deaths identified by the informants, we found that the sensitivity of the informants' network was 98% while the specificity was 99%. To measure the precision or agreement between the two sets of observations, we calculated the Kappa statistic, which was 0.93, signifying almost perfect agreement. These findings can be used to modify the methodology in the future.

### 3.2. Sociodemographic Characteristics of PRDs

The mean and median age of women who had died of pregnancy-related causes was 29 years. Three out of five women were multiparous, that is, had had more than one child, while a third were primiparous, that is, had been pregnant with their first child or had had one child. Nearly two-fifths of the deaths had taken place among women who were illiterate; a fifth were primary-educated women. Only a tenth had completed higher secondary school.

Two-fifths of PRDs had taken place among women of a lower socioeconomic status; a third had occurred among women of medium socioeconomic status and only a quarter among women of a higher socioeconomic status. To determine socioeconomic status, a wealth index was calculated for each household by running a factor analysis for 25 household amenities and consumer durable goods, energy sources, and ownership of agricultural land. This procedure is similar to that used by [22].

### 3.3. Determining Cause of Death

In disaggregating the causes of PRD, we found that 20% of these deaths were due to indirect causes and 78% due to direct causes. Nearly 2% of deaths were accidental/incidental deaths: two were due to accidental causes, one was due to homicide, and one was a suicide.

Among the direct causes, obstetric haemorrhage was identified as the leading cause of death. On disaggregating obstetric haemorrhage, we found that, in 11% of the cases, women had died due to antepartum haemorrhages and 36% due to postpartum haemorrhages. Nearly two-fifths of the deaths had occurred due to pregnancy-induced hypertension, while in 5% of the cases, death was due to abortion-related complications.

### 3.4. The MMR Estimates

To estimate the MMR for Chakwal district, we differentiated maternal deaths from PRDs by excluding accidental and incidental causes. Both the adjusted (by using the capture-recapture technique to estimate the total cases) and unadjusted numbers were used for calculating the MMR (see Table 1). The denominator, that is, number of live births, was estimated by calculating age specific fertility rate obtained from the Multiple Indicator Cluster Survey (MICS) conducted for Punjab in 2011 and the number of women of reproductive age was obtained from the district level representative data of Pakistan Social and Living Standards Measurement Survey (PSLSM) 2010-11. The adjusted MMR calculated for Chakwal district was 309 per 100,000 live births.

### 3.5. Time and Place of Death and Care Seeking Behaviour

Nearly a third of all PRDs had occurred antepartum, a quarter during the intrapartum period and up to 24 hours afterwards, and two-fifths after 24 hours but within six weeks (up to 42 days) postpartum.

Nearly two-thirds of the pregnant women documented had had more than three antenatal check-ups. More than four-fifths had reported receiving care from a doctor. Only 2% had received antenatal care from a TBA while nearly a tenth had not sought antenatal care (ANC) at all. Of the 165 maternal deaths reported in Chakwal, 37 women had died at home, 113 died at a health facility, 15 died on their way to a health facility.

### 3.6. Cost

We estimated the cost of implementing the community-based MADE-IN/MADE-FOR approach by calculating separately the logistical and administrative costs associated with the fieldwork. The cost analysis included expenditures incurred in training, salaries for enumerators, accommodation for field staff, travel, field-based monitoring, supervision, and other general expenditures (supplies and services). The per-unit cost was calculated as the per-unit WRA resident in the study area. The cost of applying the technique at the district level came to Rs 12.00, that is, $0.12 per WRA.

## 4. Discussion

MDG 5 requires a 75% reduction in maternal mortality between 1990 and 2015. To gauge if progress is being made towards achieving this goal, it is important to periodically update estimates of maternal mortality, especially at the subnational level, in order to identify specific geographic areas where interventions are required most and to assess the overall impact of existing ones. Since the 1987 Safe Motherhood Initiative was launched, new opportunities for capturing data on maternal deaths have become available, been tested, and been used in different settings.

This study has tried to assess the feasibility of applying the community-based informant technique (MADE-IN/MADE-FOR) to capture maternal deaths at the community level. We have successfully identified potential networks that could be used at the union council level—the smallest administrative unit of the government—to collect data on a sustainable basis.

According to a study carried out in India, key informants identified all births and deaths to women of reproductive age, prospectively, over a period of 110 weeks. For all deaths to women of reproductive age, they ascertained whether they could be classified as maternal, pregnancy-related, or late maternal and to do so, verbal autopsies were conducted. This low cost key informant surveillance system produced high, but plausible, birth and death rates in a remote area in India [11]. Another community-based surveillance study carried out in Cambodia assessed the performance of a community-based surveillance system that was developed to provide timely and representative information on major health problems and life events. Lay people were trained as Village Health Volunteers (VHVs) to report suspected outbreaks, important infectious diseases, and vital events occurring in their communities. Local health staff analysed the data and gave feedback to the volunteers during their monthly meetings. Over 2 years of its implementation, the system was able to detect outbreaks early, regularly monitor communicable disease trends, and provide continuously updated information on pregnancies, births, and deaths in the rural areas [10].

In most developing countries with close-knit communities, community-based networks comprising religious leaders, school teachers, and so forth already exist that have the potential to be used effectively in social mobilization activities and for collecting community-based data, provided they are properly briefed on the objectives of the endeavour and are able to appreciate the perceived benefit of their contribution to the society as a whole.

According to the last Pakistan Demographic and Health Survey (PDHS) conducted in 2006-07, the maternal mortality ratio for Pakistan was estimated at 276 per 100,000 live births. Provincial estimates were also developed. The maternal mortality ratio, per 100,000 live births, was estimated for the province of Punjab at 227; for Sindh 314; for Khyber Pakhtunkhwa 275; and for Balochistan 758. The maternal mortality ratio for urban Pakistan was 175 while for rural areas it was 319 per 100,000 live births. District level maternal mortality estimates are, however, not available. Since Chakwal district is predominantly rural, that is, 85% of the population residing in the rural areas, our estimated maternal mortality ratio of 309 (95% CI: 266–358) per 100,000 live births is close to the rural MMR estimated by the PDHS 2006-07, which shows that the results obtained from the MADE-IN and MADE-FOR methodology are according to expectation.

By introducing this methodology at the district level, we also focused on developing the capacity within the district at various levels to sustain this approach. By using local community-based networks to collect data, an additional benefit accrued was the effective sensitization of community on health issues through their leaders. For instance, the orientation provided to religious leaders has helped them develop a better understanding of women's health issues. They can now influence public opinion through their sermons and discussions with men. In the long run, these networks could be used to report additional events, such as births and incidence of communicable diseases.

**Figure 1:**
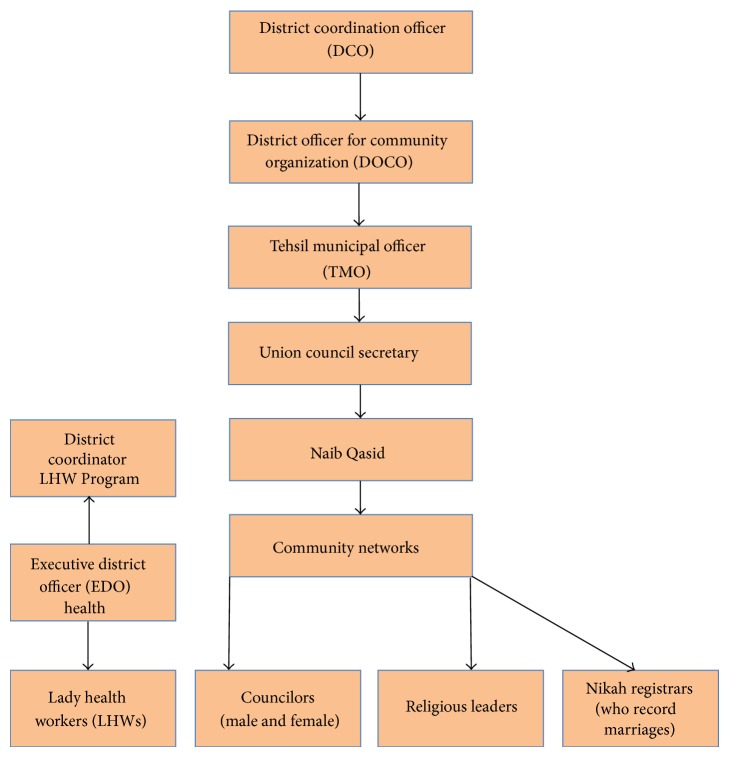
Organizational structure: from union council to district level.

**Figure 2:**
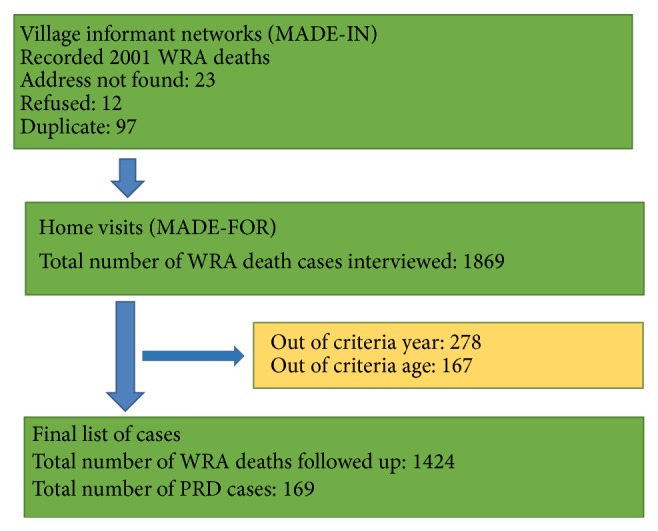
Flow of data capture.

**Figure 3:**
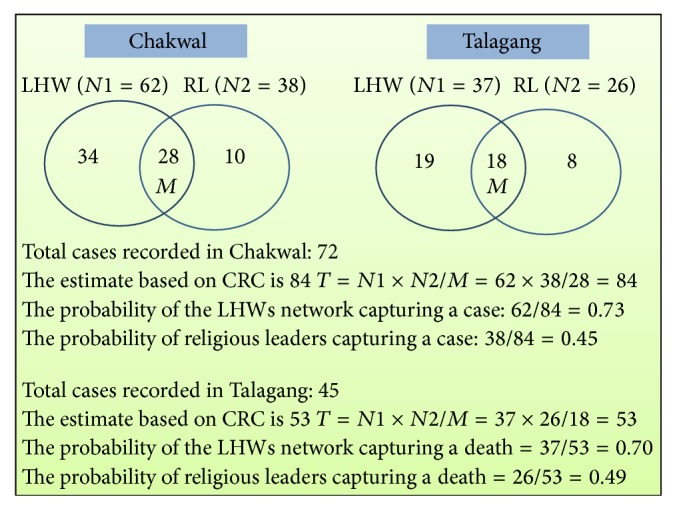
Estimating total PRDs through capture-recapture technique.

As shown in Figure 1, we used the existing administrative structure to identify the community networks so that the same networks could be used in the future as well. The District Coordination Officer (DCO) who is the administrative head of the district instructed the secretaries of the union council to identify and develop lists of religious leaders, nikah registrars (who record marriages), and councilors (elected representatives of the area). Using the already available administrative apparatus the secretary of the union council developed the lists of networks by sending out his subordinate officers called “Naib Qasids” to visit each and every village in the union council. The identified network members were given a comprehensive briefing cum training in gathering information on deaths among women of reproductive age and how to document this information. It is expected that this systematic approach can be institutionalized and can also help in improving the vital registration system. Since these networks already exist and are well respected within the communities, the approach can be sustained. The results of this pilot study were shared with the Provincial Managers who have expressed an interest in scaling up the methodology to obtain provincial level estimates.

The use of two networks allowed us to use the capture/recapture technique making the survey self-calibrating in estimating coverage as we know that a single network would not be able to capture all deaths [9].

The cost of applying this technique to obtain maternal mortality estimates at the district level was $0.12 per WRA; the cost of undertaking a survey would have been several times higher. This will allow more frequent estimates to be available to program managers to plan new initiatives and evaluate existing ones.

This study has also identified specific areas that need strong policy and programmatic interventions to improve maternal health outcomes. It confirms that maternal mortality remains a major public health issue in Pakistan and this problem may be much larger than we assumed.

The study raises important issues related to the quality of emergency obstetric care available at the facility level and the lack of a functional referral system in rural districts such as Chakwal. Disturbingly, the majority of deaths in Chakwal district had occurred at the facility level and the major causes were obstetric haemorrhage and eclampsia. Both are easily treated through simple interventions. If these women had been told where to obtain appropriate services in the first place, precious lives could have been saved.

Another aspect this study has highlighted is the suffering of poor rural women, reaffirming that economic barriers persist and affect poor women's ability to access appropriate care. In resource-constrained settings, the difference between the rich and poor in terms of ease of access to emergency care is a major determinant of poor maternal health outcomes. In most instances, these poor households are also located farthest away from emergency care [23]. The two-fifths of PRDs had been taken place among women of lower socioeconomic status and three out of five respondents reported that the cost of treatment of the deceased had been prohibitive and more than they could afford. Improving the quality of care in terms of providing at least comprehensive emergency obstetric care at the secondary tier would help poor families access appropriate care, obviating the need to travel to wide distances to access specialized care. Half of the respondents felt that arranging for funds to treat the deceased had been difficult; nearly two-thirds felt that the costs were prohibitive. These reasons may have compelled them to resort to a nearby facility irrespective of the services provided there.

Our findings are in congruence with the global evidence, which shows that most maternal deaths occur in late pregnancy to about 48 hours after delivery [24]. Therefore, policies and interventions must target this period and focus on improving active management of the third stage of labour.

## 5. Conclusion

This pilot study has added to the available evidence that shows reliable information on deaths among women of reproductive age can be obtained at a reasonably low cost from community-based networks such as religious leaders, provided that the entire district administration supports this effort and the networks realize that their work is beneficial for the society as a whole.

Finally, we would emphasize that no single approach can adequately meet all the requirements for estimating maternal mortality efficiently and with reliable precision. Complementary measurement options and opportunities such as the household census and periodic DHS surveys must remain in place to mutually validate results.

## Figures and Tables

**Table 1:** MMR estimates for Chakwal district.

	No. of live births	*n*	Unadjusted	95% CI	*n*	Adjusted	95% CI
PRMR	58522	169	289	247–336	186	318	274–367
MMR (2 years)	58522	165	282	241–328	181	309	266–358
PMDF	1424	165	12%	10%–13%	181	13%	11%–15%

95% CI have been calculated treating maternal deaths as rare events.

## Abbreviations

ANC: Antenatal care
CMW: Community midwife
LHW: Lady Health Worker
MADE-FOR: Maternal death follow-on review
MADE-IN: Maternal death from informants
MNCH: Maternal, newborn, and child health
MDGs: Millennium Development Goals
MICS: Multiple Indicator Cluster Survey
MMR: Maternal mortality ratio
MNCH: Maternal and Neonatal Child Health Program
NIPS: National Institute of Population Studies
PDHS: Pakistan Demographic and Health Survey
PRD: Pregnancy-related death
SBA: Skilled Birth Attendance
VA: Verbal autopsy
WHO: World Health Organization
WRA: Women of reproductive age.
